# Public Involvement in Cancer Research: Collaborative Evaluation Using Photovoice

**DOI:** 10.2196/75741

**Published:** 2025-07-28

**Authors:** Piotr Teodorowski, Melanie McInnes, Glen Dale, Linda Galbraith, Esme Radin, Karen Gold, Erica Gadsby

**Affiliations:** 1 Faculty of Health Sciences and Sport, University of Stirling, Pathfoot Building, Stirling, FK9 4LA, United Kingdom, 44 1786 466362; 2 Public Contributor, Stirling, United Kingdom

**Keywords:** patient involvement, prostate cancer, breast cancer, PPI, patient and public involvement, patient engagement

## Abstract

**Background:**

A public involvement group consisting of 4 public contributors with lived experience of cancer diagnosis contributed to 2 cancer research projects that focused on optimizing the diagnostic pathways for patients with suspected cancer. The public contributors have been involved from the start of the projects and were involved in aspects of the design, analysis, and dissemination alongside research and clinical teams. Despite public involvement in cancer research being seen as a key element of the research process, there is still a limited understanding of what works well and how to do it in a meaningful way for both researchers and public contributors.

**Objective:**

This study aims to evaluate the public involvement process in 2 cancer research projects.

**Methods:**

This was a collaborative evaluation with the research team and public contributors jointly evaluating the process. Data were collected throughout the lifespan of the project by public contributors through photovoice, where they collected photos that represented their experiences of involvement. At the end of the evaluation meeting, 2 separate analyses were conducted. First, public contributors reflected on their experiences using a 4-dimensional framework to capture how strong their voice was, how many ways they had an opportunity to be involved, if their feedback was implemented, and if the discussion focused on their priorities. Second, they analyzed the collected photos by organizing them alongside their narratives, explaining their meanings and comparing how they experienced the involvement process.

**Results:**

Narratives from 8 photos illustrate public contributors’ experience of involvement in these projects, presenting them in chronological order, showing how their perspectives evolved from not knowing what form the project would take, through understanding foundations and building confidence through being satisfied with the successful projects. Results from the 4-dimensional framework showed that public contributors felt that their voices were strong, and the research and clinical team mostly implemented suggested changes. The discussion focused on topics and issues that were relevant to public contributors. However, how public contributors were involved depended mainly on the research team’s decision, and they would have preferred more opportunities.

**Conclusions:**

This study has shown that public contributors can be meaningfully involved throughout the lifespan of cancer research projects. The evaluation demonstrated that establishing a strong relationship and trust between researchers and public contributors helps to ensure that the public contributors’ voice is meaningful and makes a difference in the projects. However, it also identified improvements for future public involvement. Researchers should involve public contributors as early as the funding application stage to offer more opportunities to shape research and thus have diverse involvement opportunities at each stage of the research process.

## Introduction

### Background

Public involvement has emerged as a crucial element in cancer research, ensuring studies are both patient-centered and impactful. In the United Kingdom, organizations such as the National Institute for Health and Care Research (NIHR) and Cancer Research UK mandate public involvement in funded research to ensure transparency and accountability [1,2]. By actively engaging patients, caregivers, and community members in every phase of a study, from identifying research questions to designing research methods and disseminating findings, the public voice introduces unique insights that extend beyond traditional clinical or academic perspectives [3,4]. Furthermore, public involvement builds trust between researchers and the communities they serve, leading to improved recruitment, better retention of study participants, and ultimately more meaningful outcomes that reflect real-world needs [5]. Despite these acknowledged benefits, systematic evaluation of public involvement processes and their impacts on research outcomes remains a critical area for further investigation.

We follow the NIHR in the UK definition of public involvement in research as research “being carried out ‘with’ or ‘by’ members of the public rather than ‘to,’ ‘about,’ or ‘for’ them” [6]. It is the active partnership between researchers and members of the public, who work collaboratively throughout the research process rather than merely serving as study subjects [7]. These definitions are operationalized by the UK National Standards for Public Involvement, which provides a framework for ensuring that engagement is meaningful, inclusive, and sustainable [8]. The underpinning principles of respect, support, transparency, and accountability have been central to our approach, guiding the integration of public perspectives throughout the research process.

While public involvement is widely regarded as beneficial in cancer research, the literature reveals significant challenges in ensuring that such involvement goes beyond superficial consultation. Scoping reviews in health research indicate that public involvement activities are predominantly advisory and tend to occur during the early stages of research, thereby limiting the influence public contributors have on key decisions [7,9]. Although researchers value public input, integrating lay perspectives on an equal footing with academic expertise can be challenging. Structural aspects, such as the hierarchical nature of academia and the research environment, pose additional barriers to sharing power and influence [10]. Resource constraints further compound these issues; without adequate time and funding dedicated to public involvement, the process can be rushed and superficial, undermining meaningful involvement [4]. Overall, the literature emphasizes the need to continuously reflect on power dynamics, proactively plan public involvement, and create environments where public contributors have genuine influence.

Critical reflection of public involvement from both public contributors’ and researchers’ perspectives remains limited in the literature. As effective partnerships require careful attention to process, robust evaluations of the processes and outcomes of public involvement are essential [11]. However, many projects do not formally assess the impact of public involvement or report the challenges encountered. Reflecting on both successes and difficulties is critical to strengthening public involvement practice and ensuring that public contributions are genuinely integrated into research. In this context, evaluative frameworks, such as the 4-dimensional theoretical framework developed by Gibson et al [12,13], are invaluable for guiding the evaluation of public involvement in cancer research. We present an evaluation of the involvement of 4 public contributors across 2 cancer research projects.

### Background to Evaluated Cancer Research Projects

The public contributor group has been established to support 2 Cancer Research UK-funded projects [14]. They both aimed to optimize the diagnostic pathway for patients with suspected cancer and were conducted in partnership with NHS Fife and NHS Forth Valley in Scotland. The project in Fife introduced the nurse-led diagnostic clinic for suspected urgent prostate cancer referrals. The Forth Valley project implemented direct referrals to secondary care for urgent suspected breast cancer by removing primary care appointments. Both projects, which ran simultaneously, used a hybrid effectiveness-implementation study design with similar research methods, focused on health outcomes, patient experience, costs to the health care system, and the process of implementation. Therefore, the decision was made to share experiences between these 2 projects and organize joint management and governance groups such as this one for public contributors. This paper refers to both breast and prostate interventions as the projects.

The research team comprised academic and clinical researchers with backgrounds in public health, health services research, psychology, and oncology, all with a shared commitment to inclusive, person-centered research. The team had previous experience in participatory methods and a belief in the value of lived experience as a form of expertise. These perspectives shaped the approach to involving the public as active partners and informed the interpretation of findings with a focus on equity, collaboration, and real-world relevance.

Public involvement guidance recommends involving more than 1 public contributor in research [6]. In these projects, the research team decided to involve 4 public contributors. This was based on previous experiences that small groups offer an opportunity for all public contributors to share their opinions, but also to bounce ideas through peer discussion and support. Previous cancer projects showed that 2 [15] or 3 public contributors [16] can meaningfully influence research.

The projects were launched in May 2023. In July, the research team recruited 4 public contributors with lived experience of breast (n=2), prostate (n=1), or another form of cancer (n=1) to the public involvement group. One of them was previously involved in research as a public contributor. None of the public contributors knew each other or the researchers before their involvement. The public contributors were not representative of the general population but rather emphasized the diversity of experiences [17]. This diversity included, for example, previous research experience, type of cancer, age, and whether cancer was diagnosed among other family members. Identifying potential public contributors can be challenging, and engaging with established contacts and cancer charities can facilitate that process [18]. They were recruited through established contacts, charities (eg, Breast Cancer Now), and the 1000 Elders Group at the University of Stirling [19]. Interested members of the public contacted the researcher with a short paragraph about why they were interested in the projects and subsequently met with the researcher to discuss the opportunity further. This meeting was not an interview process, but rather an opportunity for interested individuals to learn more about the projects, the role involved, and the time commitment required to make an informed decision about becoming a public contributor.

Public contributors contributed in 2 ways. First, they attended management group meetings alongside researchers, clinical teams, and funder representatives. The purpose of the management group was to oversee and manage projects, ensuring the cross-fertilization of ideas across projects and enabling input. These meetings took place approximately every 3 months in person or hybrid. Second, the public involvement group met regularly with the research team. These meetings were online. One researcher was identified as a contact for public contributors through email, and peer support was offered through WhatsApp. Public contributors received £20 (US $27.5) shopping vouchers for attending meetings and were offered reimbursement for travel expenses (public transport or mileage).

During the design stage (May-July 2023), public contributors were introduced to the projects, the implementation plan, and the draft methodology and agreed to terms of reference for the public involvement group. Terms of reference defined the role of public contributors in the projects and their responsibilities (eg, maintaining confidentiality of all project-related information). It also stated the researchers’ responsibilities, such as providing support, training, and offering regular updates on the progress of the projects and remuneration process. The terms were presented as draft versions and jointly agreed upon by both sides. Later, public contributors co-developed the theory of change (mapping how each intervention was expected to lead to desired outcomes), capturing patient perspectives and ensuring that the language was inclusive. They also reviewed and revised draft data collection tools and participant recruitment plans. This ensured that all questions in the interview guides and patient questionnaires were written in lay language and easily understandable by members of the public. Public contributors provided additional questions to capture the patient experience or reordered the question order for clarity. The protocol detailing the projects has been published, and public contributors have co-authored it [14].

During the implementation (August 2023-July 2024) and write-up stage (August- November 2024), public contributors acted as critical friends during the management group meetings as both clinical and research teams updated on the projects’ progress. During meetings with the research team, they were involved in the initial analysis of staff and patient interviews. This was facilitated by training on how to be reflective when interpreting qualitative data. Thus, public contributors started to reflect on how their experiences impacted their interpretation of data. Table 1 summarizes reflective questions asked around each data segment shared with public contributors. The research team shared initial interviews as the data collection was ongoing, public contributors contributed not only to analysis (eg, developing codes during qualitative analysis) but also identified follow-up questions for future interviews. Later, public contributors were involved in writing policy reports and lay summaries.

**Table 1.** Reflective questions shared with public contributors during qualitative data analysis.

Reflective question	Aim of this question
How does this quote make you feel?	Offered space for a public contributor to reflect on the segment and link it with their experience.
Did anything surprise you and why?	Public contributors linked the situation in the segment to the theory of change and assumptions underpinning the project implementation.
Do some parts of this discussion benefit from follow-up questions?	Critical friend feedback if the interviewer potentially captured all relevant information from the participants in that part of the interview.
Should we explore another issue with participants – staff or patients?	Identified areas for future interviews that should be explored.
How would you describe what is going on in this quote?	Public contributors captured keywords that were later used to develop codes for the coding process in NVivo (Lumivero).

The research team and public contributors have developed a close working relationship throughout the research projects to facilitate the evaluation process. Researchers provided a nonjudgmental space and encouraged discussion [20]. This was achieved by having jointly agreed ground rules for each meeting (including evaluation) centering around listening and respecting each other’s points of view and being a safe space to share perspectives and experiences. Honesty was crucial to developing trust [21]. Public contributors were constantly kept updated on the research progress through the management group and email updates. It takes time to develop the relationship between researchers and public contributors, but continuous involvement between the same people can facilitate that process [20]. In these projects, public contributors engaged with two key researchers for an 18-month period, thus establishing that connection.

Public contributors attended the Scottish Cancer Conference. One public contributor co-presented with the researcher on the public involvement process alongside initial findings from this evaluation at the Patient and Public Involvement Event. The research team invited public contributors to a thank-you dinner to celebrate the end of the projects.

### Evaluation Aims

This paper presents the evaluation of our public involvement process in these 2 cancer projects. The objectives are to: (1) capture the involvement from the perspective of public contributors, (2) understand interactions between public contributors and the research team, and (3) identify what worked well and areas for improvement.

## Methods

### Design

The decision to evaluate the public involvement in these projects was undertaken jointly by the research team and the public contributors’ group in July 2023. It was agreed to be a collaborative evaluation, thus actively engaging public contributors in the evaluation process. Before co-authoring this paper, all public contributors became involved in data collection, analysis, interpretation, and reporting [22]. Principles of collaborative evaluation are similar to, and often used simultaneously with, other participant-oriented evaluation approaches (eg, participatory and utilization-focused evaluation) [22]. These, in turn, show similarities to public involvement in research [23]. Table 2 summarizes the key features underpinning this evaluation. To further ensure the quality of this paper, the Standards for Reporting Qualitative Research [24] are reported in Checklist 1.

**Table 2.** Key features of the collaborative evaluation adapted from O'Sullivan [22].

Aspects of collaborative evaluation	How incorporated in this evaluation
Primary evaluation focus	The evaluation aimed to improve future public involvement run by the research team, and those where public contributors would participate.
Evaluation decision-making	Public contributors themselves chose the level of involvement.
Stakeholder roles	Public contributors were partners in the evaluation process and data sources.
Evaluator roles	Roles agreed. PT took the role of the evaluator as the team leader in the evaluation and public contributors were involved throughout the process.
Pre-evaluation clarification activities	Public contributors discussed and agreed on the evaluation aims.
Design	The evaluation was systematic and rigorous, with the process agreed upon at the start of the projects.
Type(s) of data collection	How public contributors’ experience was captured was collaboratively agreed upon at the start of the evaluation process.
Type(s) of data reporting	Reporting has been collaboratively agreed upon between the research team and public contributors. Public contributors decided which collected data would be presented in the paper.
Evaluation capacity building	Public contributors learned new skills and became more confident researchers by being involved in the evaluation (alongside skills learned in the projects).
Cultural responsiveness	The research team had previous experience in organizing and evaluating public involvement activities.
Systems and networking considerations	Learnings from the evaluation were used to feed into the broader public involvement strategies and future research projects. This included dissemination of evaluation findings.
Active stakeholder engagement in evaluation implementation	Public contributors were actively involved in all stages of the evaluation and co-authored or co-presented any findings arising from the evaluation.

### Data Collection

The collaborative evaluation was supported by creative research methods. Creativity has been used successfully in previous public involvement [25] and to evaluate the involvement process [26]. Creativity can offer public contributors a space to reflect on sensitive topics and express in their way their understanding of the world [27], thus providing insights that otherwise would be missed.

Public contributors had the option to choose between two creative methods. First, photovoice [28] and second, photo-elicitation [29]. Both methods entailed public contributors sharing their experiences through photos; they used the photos to symbolize their experiences and described how the images expressed their experiences. In photovoice, public contributors would take and bring their own photos, whereas, in photo-elicitation, the research team offered a choice of images. The photovoice method has been ongoing throughout the project, whereas the photo-elicitation method was available during the evaluation meeting. However, public contributors decided not to use any photos the research team offered; instead, they chose only those they took themselves. Photovoice and photo-elicitation were selected as creative methods due to their scope to capture people’s experiences in an empowering way, where public contributors could actively shape data collection and offer space for reflection (both on individual and group levels).

Photovoice methodology guidance outlines 9 steps for using the method, although there is flexibility in how to implement it in each study [30]. Table 3 summarizes how photovoice was approached in this evaluation.

**Table 3.** Photovoice steps as outlined by Wang [31].

Photovoice steps	How was it implemented in this evaluation
Select and recruit a target audience of policymakers or community leaders.	Photographs should be shared externally to influence policymakers. The decision was made to share these through academic conferences and peer-reviewed papers targeting those working in cancer research (both researchers and public contributors). Public contributors and researchers co-presented the photos during an academic conference and co-authored this paper.
Recruit a group of photovoice participants.	All public contributors had an opportunity to take part in the photovoice.
Introduce the photovoice methodology to participants and facilitate a group discussion.	Researchers organized a training session on what photovoice is, how photos could be used to describe experiences, and explained the key rules of what kind of photos could be taken. Public contributors were reassured that this is not about becoming professional photographers.
Obtain informed consent.	All public contributors received a consent form and a participant information sheet before taking part in the photovoice. This was separate from the terms and conditions of being involved in the projects as public contributors.
Pose an initial theme for taking photos.	During some of the meetings, the research team brought photos, encouraging public contributors to choose one that represented their experience of the discussion held. This helped to build confidence in how to use photos in the discussion.
Distribute cameras to participants and review how to use them.	Researchers checked if all public contributors had access to a smartphone camera or a professional one.
Provide time for participants to take photos.	Public contributors could take photos anytime throughout the projects and share them with the researcher, who uploaded them to the secure document accessible by all public contributors.
Meet to discuss photographs.	The photographs were analyzed during the evaluation meeting. The discussion helped to establish consensus on how photos represent public contributors’ experiences.
Plan with participants a format to share photographs and stories with policymakers or community leaders.	Co-presenting at a conference and co-writing this paper.

### Analysis

**Figure 1.**
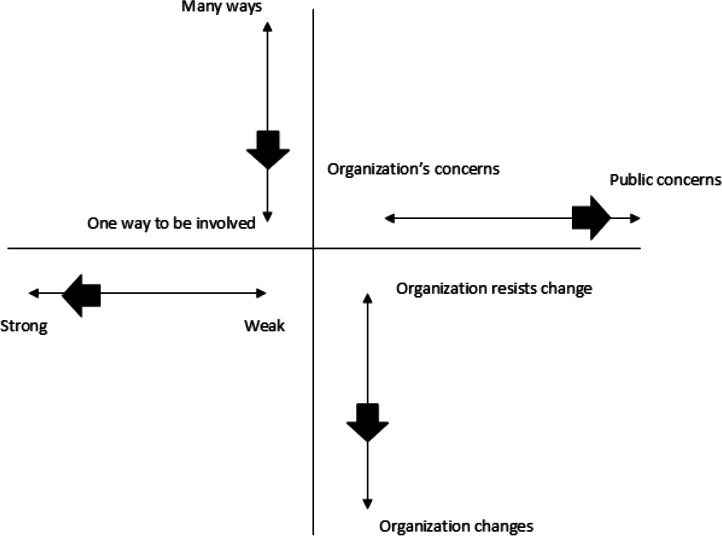
Mapped findings within the 4-dimensional framework as based on work by Gibson et al [12,13].

At the end of the projects, PT, MM, and the public contributors met online on Microsoft Teams to evaluate the involvement process using the 4-dimensional framework [12,13]. The framework was chosen as it offered a comprehensive assessment of the public involvement interactions and captured subjective perspectives among public contributors. Researchers and public contributors had different perspectives of involvement (as those involving and being involved); there were also differences within the groups, as 1 researcher had experience of public involvement in research, as well as 1 public contributor. The four dimensions of the framework are: (1) the strength of public contributors’ voice, (2) the number of ways they were involved, (3) if the research team implemented change, and (4) if the focus of involvement was on public contributors’ priorities or the research team’s. Each dimension was discussed in turn, and experiences were mapped as a group consensus. As the meeting took place online, PT shared his screen during the mapping exercise. PT and MM took notes from the discussion. However, all public contributors had an opportunity to deviate from the group if they wished. The process consisted of public contributors placing themselves on a line with two opposite ends (eg, weak voice vs strong voice in projects) to explain why they had placed their experiences there. The findings were presented as a diagram (see Figure 1) and a narrative way.

Collected photos were analyzed separately, discussed, and organized in a way that presented the joint experiences of being involved in the projects. Each photo was presented, and the author shared the story behind it, which led to questions from other public contributors and a reflection on how contributors' own experiences related to the one presented. Thus, this offered a more comprehensive understanding of public contributors’ experiences. All public contributors agreed jointly on the interpretation of the data through the discussion. Investing time in building trusting relationships from the beginning and throughout the project was helpful for mitigating potential biases, such as social desirability bias and researcher influence during the evaluation meeting.

### Ethical Considerations

The ethical approval for this evaluation has been granted by the University of Stirling General University Ethics Panel (15773). The ethical approvals for projects have been granted previously by South Central—Oxford A Research Ethics Committee (NHS Fife, 23/SC/0252) and by East of England—Cambridge East Research Ethics Committee (NHS Forth Valley, 23/EE/0168). As only 4 public contributors participated in the evaluation and they co-authored this paper (alongside other dissemination outputs), their confidentiality was limited. This was discussed with them beforehand and explained in the consent form that all public contributors signed before the start of the evaluation. Participation in the evaluation meeting was reimbursed with a £20 (US $27.5) shopping voucher, in accordance with the established policy for any type of meeting attended by public contributors.

## Results

### Photovoice

Findings from the photovoice method are presented and discussed chronologically to show how public contributors’ experiences with projects have evolved over time. A total of 3 public contributors shared 8 photos throughout their involvement. The public contributor who did not share a photo felt that their experiences were already captured in others, as data collection was ongoing and everyone had access to already shared photos. The group discussed these jointly, and a consensus was reached to share all of them as joint experiences of their involvement.

**Figure 2.**
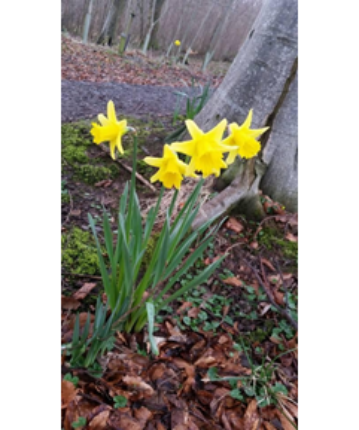
Photo shared by public contributor.

At the start of their involvement (see Figures2  3), public contributors had to visualize what these projects would look like and what their role would be in this process. Inductions and support from the research team were essential, and building connections with other public contributors was key in establishing the public contributors group.

**Figure 3.**
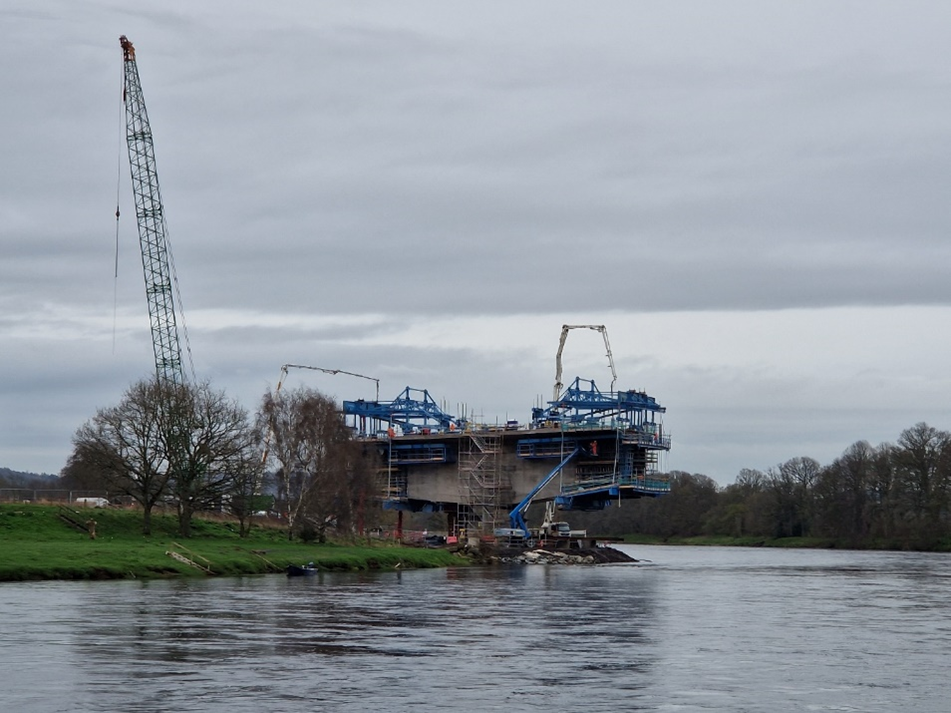
Photo shared by public contributor.

The daffodil facing outwards is the thought process, visualizing how the project will form. The little bunch of daffodils standing tall reflects that vision coming to fruition with our now formed group!

[Figure 2]

I find this photograph of the building of one of the pillars of the Cross Tay road link similar in concept to our project at the moment.

The foundations have been firmly set and we are now reaching out to complete the project.

Once finished many people will benefit from this. There is however still a lot of significant work to do.

[Figure 3]

**Figure 4.**
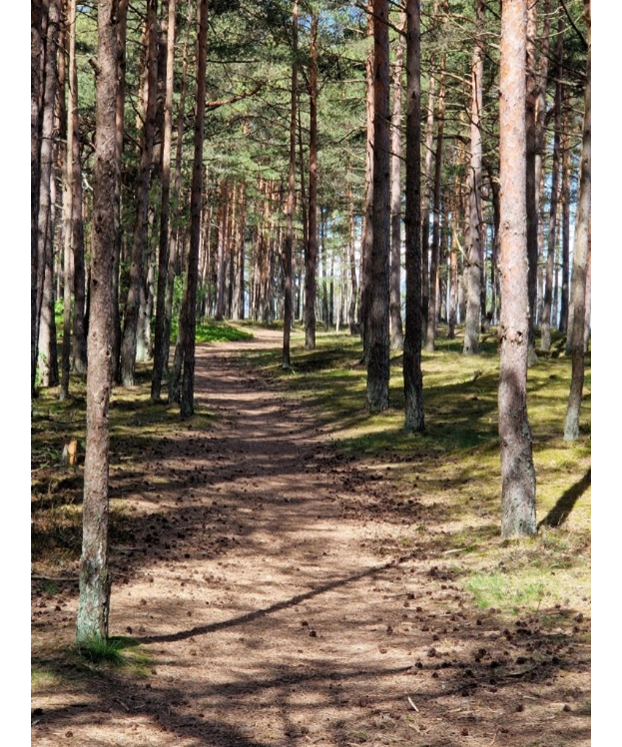
Photo shared by public contributor.

The projects focused on patient diagnostic pathways, and public contributors felt that the path through the woods appropriately represented the pathways being developed (see Figure 4). The imagination of a forest and an uncountable number of trees looked overwhelming—similarly, project plans, expected outcomes, and how these would be delivered felt overwhelming. However, public contributors agreed that the path and direction became more apparent as the projects progressed.

Here is my Photovoice image from Jurmala, Latvia, following our last project conference call:

Sometimes it is difficult to see the “wood from the trees” from information collected but this image reflects to me that patient feedback, from both the Forth Valley & Fife projects, shows us that we are on a good clear path forward.

[Figure 4]

The next picture could not be included as it shows people queuing to see a rare type of plane arriving at an airport, and we were unable to obtain consent from those pictured. This waiting for the plane described the public contributor’s feelings toward the project. This includes excitement about the change in health care (the new cancer pathways) and uncertainty about when it will fully be implemented and what it will bring to patients. Waiting in the queue also offered a space for reflection on the many small steps and pieces that need to come together before implementing a change and all the components required to achieve the intended outcomes. This was likened to the necessary procedures required for landing a plane (control tower, weather, runway, pilots, landing equipment, etc). In a way, the people queuing mirrored the progression of the project.

I like the image of people waiting by the road- not sure when they will get what they want/need, but an overarching structure is in place to ensure it will arrive at the right time. […] People are anxious to see the plane but it is controlled by a system/pathway that will determine the landing time.

**Figure 5.**
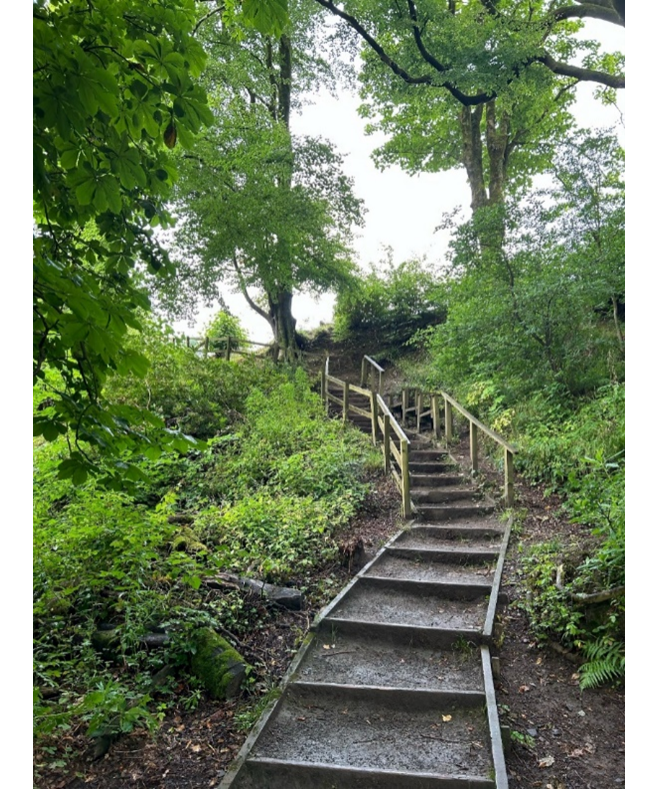
Photo shared by public contributor.

Being involved in the research was like climbing on steps that had no handrails, so in turn, this approach could be a wobbly climb to start with, but as the public contributors reached the middle, they saw the handrails offer confidence and assistance to make the climb to the top safely achievable (see Figure 5). Being involved in the project felt like a wobbly climb, filled with uncertainty about whether things were heading in the right direction. Each handrail represented the meetings with the project team, where assistance and feedback guided the way. The middle ground of the project has been reached successfully, and there was confidence that the team would achieve the high point (expected outcomes). Public contributors reflected that the collaboration and feedback from the research team acted as stabilizing support, helping the project move forward confidently.

On approach to the steps we see no handrails so in turn could be a wobbly climb to start with but as we reach the middle we see the handrails offer confidence and assistance to make the climb to the top safely achievable.

Thought about the group being formed at the beginning, we have made the middle ground successfully and confident we will reach a high point.

[Figure 5]

**Figure 6.**
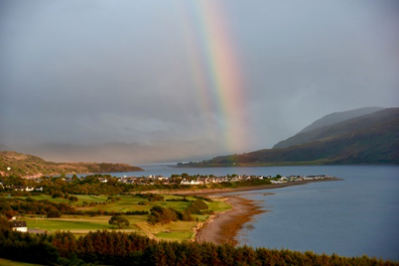
Photo shared by public contributor.

Public involvement in these research projects had moments of hope in terms of project outcomes helping those going through the cancer diagnostic journey; the following photo of the rainbow (see Figure 6) symbolizes positivity and high expectations for the success of the project. Public contributors expressed their surprise and appreciation for the staff’s (clinical, management, administrative, and general practitioner reception teams) reactions to the suggested changes coming from public contributors. They noted their appreciation for the staff’s positivity and adaptability in creating a more patient-centered process and how most staff embraced the change in the pathways.

Here is my Photovoice image on approaching the end of our project together. This photograph was taken recently overlooking Ullapool.

Seeing the rainbow shining over Ullapool amongst the grey background reminds me of the hope that our project will give a better journey to those who have to experience the process of cancer tests and treatments.

[Figure 6]

**Figure 7.**
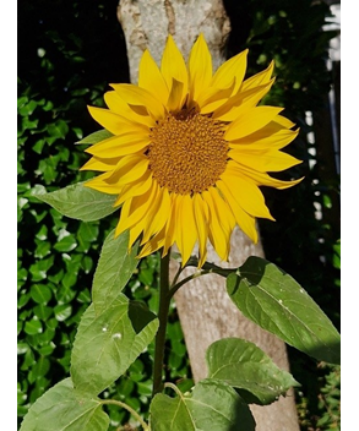
Photo shared by public contributor.

The sunflower photo (see Figure 7) symbolizes the joy of contributing to a successful project. This focused on both personal pride in contributing to these projects and the perception that, thanks to their involvement, the projects have been executed well.

This image reflects the joy in having contributed to a successful project. I am sure future patients will benefit from the changes made.

[Figure 7]

**Figure 8.**
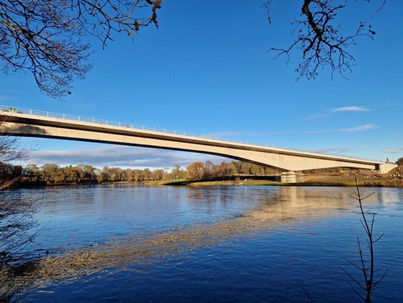
Photo shared by public contributor.

The final photo (see Figure 8) is the repeated image of the same bridge, but now completed. The simplicity of the finished bridge is the result of 2 years of meticulous planning, collaboration, and hard work, and is thus like the project’s development. The functional purpose of the bridge, connecting people and enabling smoother travel, mirrors the project’s aim of optimizing patient pathways, ensuring patients can access care more effectively and efficiently. The bridge metaphor emphasized the importance of the people and resources that contributed to the bridge, which parallels teamwork and collaboration within the projects. Public contributors agreed that the images of the bridge, from its early stages to completion, were a fitting representation of the research process and thus captured the essence of their involvement.

The Cross Tay Project has mirrored our project being built in exactly the same timeframe.

Earlier I sent in a picture of this bridge in progress drawing similarity to our project in that once successfully finished it would provide benefit to patients for years to come.

Both this bridge and our project are now almost complete and will successfully improve the lives of their customers/patients.

Congratulations on a successful project.

[Figure 8]

### The 4-Dimensional Framework

#### How Strong was Your Voice When Being Involved as a Public Contributor in These Projects (Weak Voice or Strong Voice)?

The public contributors felt that they had a relatively strong voice in terms of raising critical questions and that their questions were well received by both the research team and health care professionals. However, they felt that, as they had not seen the final report, they were not sure how their voices had impacted the projects (reports were being peer-reviewed during the time of this evaluation). They noted that they could have received more regular updates, especially regarding whether their feedback had influenced project activities.

They acknowledged the constraints of a project that was already planned and underway before they joined as funding was secured. The public contributors sometimes questioned where their voices could reasonably be integrated.

#### How Many Ways Did You Have an Opportunity to be Involved in These Projects?

Public contributors felt they could have been involved in more ways and would have liked more opportunities to get involved. There were relatively few ways to be involved, and these depended on activities organized or offered by the research team, thus being a top-down approach. They felt they could have contributed better if they had been involved earlier in the project, during its development, so they could have a stronger voice in its development and choice of methods. Public contributors expressed a willingness to take on a more active role in the projects and suggested that they could have contributed more if additional opportunities had been offered.

#### Was the Involvement Focused on the Organization or Public Concerns?

Public concerns were at the forefront of the projects. The focus was on issues that were important to public contributors. These included the well-being of those going through a cancer diagnosis, understanding the experiences of past and present patients, and wanting to provide more patient-centered and faster diagnosis to all those potentially going through the pathways.

There was recognition that certain issues were more relevant to the research team or clinical staff; however, it was felt that these did not take over the agenda of meetings attended by public contributors.

#### Has the Organization Changed or Resisted Change Based on Your Feedback?

Public contributors perceived that the research team had listened to them, and that their feedback was taken on board (or at least they did not hear back that it was not beneficial to the projects). However, as there was limited communication, the public contributors ranked the strength of their voice to implement change as fairly weak, but still, the research team and health care professionals implemented change. Public contributors suggested providing more regular feedback to keep them informed about the outcomes of their contributions.

Other limitations were around what things public contributors could comment on, as these were limited to mostly patients’ data and experiences. Routine data and economic analysis became available toward the end of the project, and public contributors did not have the opportunity to discuss the findings with the research team or the implications of these findings on patients.

Public contributors felt that sometimes healthcare professionals’ perspectives were heard more—they did not expect to have a strong voice, but it was the feeling that their voice carried louder. For example, the research team presented data when preliminary analysis took place, and data collection was still ongoing. This was seen as relevant to comment, but they also felt that it would not substantially change the projects. On the other hand, the research team met regularly with health care professionals (in some cases weekly) to discuss how the projects progressed. Meetings with public contributors were part of a 3-monthly management group meeting or as required when new findings were available.

## Discussion

### Principal Findings

This paper presents an evaluation of the experiences of 4 public contributors with lived experience of cancer in 2 cancer research projects. The creative methods allowed the capture of public contributors’ experiences throughout the project; these were revisited during the evaluation meeting, thus offering a critical space for reflection and identifying what worked well and areas for improvement. The findings offer new insights into the process of involving public contributors in cancer research projects, but the findings could also benefit those working in public involvement in other fields.

Developing working and trusting relationships with public contributors requires time [21]. None of the public contributors involved in the projects or researchers have worked together previously. However, introductions and relationships were developed from the start (eg, through the WhatsApp support group). Remote involvement became commonplace during COVID-19 [32], but in-person meetings and attending conferences together built further connections among public contributors and with the research team.

Regular communications from researchers make public contributors feel a part of the team and foster productive relationships [33,34]. Feedback on how public contributors’ comments were incorporated into the work could have been provided more regularly. Some attempts to incorporate a “you said, we did” approach were made at the start of the work. As this was time-consuming, the research team prioritized discussion of the projects rather than coming back to already discussed (and implemented input). These limited involvement opportunities were primarily affected by the restricted budget for reimbursement. Providing satisfactory feedback is not a new challenge in public involvement [34], and the expectations should have been agreed upon early in the projects and regularly revisited.

One of the functions of public involvement in research is to make it more democratic [35]. Cancer research is often embedded in complex health care settings, so training and support offered during the introductory stage of the involvement process increased the public contributors’ ability to shape the projects. Through membership in the management group, public contributors had an opportunity to assess their influence over the research project in the same way as other stakeholders. However, early involvement of public contributors from as early as the prefunding stage can help to develop stronger relationships and facilitate a stronger public voice [33]. Recruiting public contributors after the projects were designed meant that there was limited scope for them to influence the overall design of the projects and research methods. Being a part of the process from the funding application stage could have made a difference, for example, by including public contributors in the preapplication study design and as the co-applicant on the funding application [36].

Similarly, the budget limited how much researchers could have involved public contributors at each stage of the projects. Thus, public contributors were willing to do more work, and they felt that they could commit to more and would prefer to receive documents in advance to read, not just during the meeting. This is not an unexpected finding given the nature of health services research, where the need for pragmatism often limits the depth of public involvement due to resource, time, and organizational constraints [37,38]. However, the level of public contributors’ involvement, though contributing to some analysis and dissemination, seemed to be higher than most of the cancer research projects, as the scoping review by Colomer-Lahiguera and colleagues [7] found that it is rare for cancer researchers to involve public contributors outside design stages.

### Public Contributors’ and Researchers’ Reflections

The following is a reflection from public contributor GD:

Having experienced prostate cancer diagnosis and treatment, it was a privilege to be given the opportunity to participate as a public contributor.

I was keen to give something back to the health service but unsure how to do this without a medical background. This project showed me both how much work is ongoing to improve the patient experience as well as how public volunteers can contribute their own experience into projects. The project team were very accepting of our comment and feedback and we had several in-depth discussions around the project scope and then on the subsequent patient feedback.

Throughout the project, I gained confidence that I could contribute value to the project. I was so pleased that my life skills in another profession could be used in a medical research study. Being an amateur photographer I also enjoyed contributing to the Photovoice by choosing and photographing a subject that reflected on how I saw the project progressing. This was a new tool to me and one I enjoyed contributing to.

It was a pleasure to contribute to this project and I am now confidently looking forward to participating on future projects.

[GD]

The following is a reflection from public contributor KG:

Having had my own breast cancer diagnosis alongside family members with both breast and prostate and as a mum of three naturally worry about their future health I became interested in ways of being able to find out more about screening diagnostics, treatments, trials that could be evolving therefore getting involved in a PPI group seemed the perfect way of gaining knowledge when I didn’t have a medical background.

Both studies were very interesting for me as a patient contributor. I enjoyed engaging in the in person meetings with the research team and health professionals where I was able to gain in depth knowledge, contribute and felt listened too. In the beginning I wasn’t sure what value I would be able to bring to the group but to see some of those suggestions that myself and the PPI put forward actually implemented into the study along the way really confirmed to me how valuable a part we played.

Over the 18 months it was encouraging to see each step progress within both studies and looked forward to hear feedback from medical professionals and patients at our meetings which was reassuring to see it was making a valuable difference to the patients but also see and hear the enthusiasm within the medical teams implementing this study.

I also enjoyed using the Photovoice tool throughout the project.

Having never delved deeper into photos other than it being a pretty picture to then be able to interpret it into a personal reflection of how I saw the study in its various stages and sharing all of our reflections within the group was really enjoyable and something I intend to keep doing with photographs, a new hobby perhaps.

This study has given me the confidence to seek out other projects I could become involved in and was my small way of giving something back to the NHS who were and still are invaluable to me.

I have met some really inspiring individuals within this project and I hope our paths will cross again.

[KG]

The following is a reflection from public contributor ER:

I worked in the clinical sector for many years and had to retire due to ill health. I was very happy to be able to help with the study the team were working on as being a cancer patient I could see the very many advantages for the patients of the study and how it helped to reduce the stress of waiting for appointments. The fact that the patient could be immediately referred to the hospital breast or prostate cancer clinic rather than wait to see a GP was to me such a benefit.

I am well aware what the stress of waiting to see clinicians affects patients and this study was to me a breakthrough for the patients.

The team at Stirling University were so welcoming and friendly but at the same time professional in running the study with many updates and online meetings. I especially enjoyed the face to face meetings when the whole team including the clinical staff met together to discuss and hear updates and results of how the patients felt about the study.

[ER]

The following is a reflection from public contributor LG:

I started work on this project 18 months ago. It was the first pathway project I had been involved with. I have been Co-Investigator in a number of studies—with involvement from design, pre-clinical to dissemination, but this was usually centred around clinical trials or occasionally funding and strategy.

I was keen to be involved with a pathway project—I believed that they could have a significant impact for patients, and early evidence for this study suggests that this may be the case.

For me, the process was very enjoyable—staff were extremely supportive and helpful—in PPI, you can find yourself being there to tick a box. This was not the case in this study—we were fully involved, and our thoughts and opinions were met with considered feedback. The PPI group worked well together and are hopeful we can work together again with future studies.

Our feedback and evaluation was captured using a variety of measures, which, for me, was a learning experience itself.

My conclusion on the study itself is that the importance of pathway studies is often underestimated. This is potentially a way to improve patient and staff experience in a way that should fit within NHS budgets.

A very positive and enjoyable experience.

[LG]

The following is a reflection from researcher MM:

Engaging public contributors soon after project inception transformed our approach and deepened our understanding. In early meetings, GD, KG, ER, and LG questioned our assumptions about diagnostic pathways, pushing us beyond purely clinical and academic perspectives to focus on lived experience. Their use of Photovoice, selecting and discussing images that represented different stages and experiences of research involvement, revealed perceptual and practical challenges that we could have overlooked. In turn, we became more open about evolving data, acknowledged uncertainties, and truly listened. I came to appreciate that public contributors are essential partners whose insights enhance relevance, feasibility, and impact. This collaboration will fundamentally shape how I design, conduct, and disseminate all future research.

[MM]

The following is a reflection from researcher PT:

Public contributors’ comments made me perceive the research projects differently. For example, when we shared initial interview data with them, public contributors noticed issues and had priorities different from some of the initial thoughts in the research team. Thus, their feedback shaped the analysis. I feel that themes became more patient-focused—something that would not have been possible without their genuine involvement.

From the start, I wanted to ensure that public contributors feel a part of the team and have an enjoyable experience. Public involvement should not be about a one-off activity but rather a long-term process. Informal engagement was one of the ways to achieve it. First, the WhatsApp group became a good space for conversation. Second, I actively looked for opportunities for public contributors to attend conferences that were related to cancer research. This in-person networking helped to share ideas and get to know each other better, leading to more trust. That trust was an essential part of the evaluation process as public contributors felt comfortable telling us what went well and where the research team could have done better. For me, some of their feedback was expected, but other comments were surprising, for example, that they wanted to be involved in more ways than were offered. This is a meaningful learning that I aim to apply in future public involvement.

I plan to stay in touch with all public contributors, and we hope to invite them to new involvement opportunities in the future.

[PT]

### Strengths and Limitations

One of the strengths of this evaluation was the active involvement of public contributors from the design through analysis and reporting. There have been calls to evaluate public contributors’ experiences of co-authoring publications and sharing the findings [39]. However, public involvement evaluation tools have been previously criticized for rarely being designed to report back findings to public contributors [40].

Photovoice allowed for a unique space to discuss the public contributors’ experiences. This method was agreed at the start; however, 1 public contributor (who shared 1 photo) reflected later that they had difficulty interpreting images due to dorsal stream disruption and suggested that auditory descriptions or sound clips could be more accessible and helpful in representing their experiences. Another public contributor did not share any photos. Future projects using photovoice should embed introductory photography training to support people’s confidence and increase the number of photos collected [41].

The perspective of this evaluation was only on public contributors’ perspectives as they collected photos. However, during the evaluation meeting, public contributors felt that they would like to see some photos from researchers to understand other perspectives on how public involvement worked in these projects. The evaluation plan was designed at the start of the research projects, and it was agreed with the public contributors. The aim was to collect data around public contributors’ experiences. As data collection was ongoing throughout the research projects, it was not possible for researchers to take photos retrospectively. Future evaluation should ensure data collection from all groups to capture the involvement process from researchers’ and public contributors’ perspectives [26].

### Conclusion

This paper presents an evaluation of public involvement in 2 cancer research projects. The evaluation identified what worked well and areas for improvement, especially around building relationships and actively involving public contributors throughout the lifespan of the project. Public involvement in cancer research is still developing, and this evaluation has provided evidence of how to do it in a meaningful way for both the researchers and public contributors. Future cancer research projects should ensure public involvement as early as possible and provide a wide range of involvement opportunities for public contributors.

## Supplementary material

10.2196/75741 Checklist 1 Completed Standards for Reporting Qualitative Research (SRQR) checklist.

## Abbreviations

NHS: National Health Service
NIHR: National Institute for Health and Care Research
